# The Role of Intermittent Fasting in the Management of Nonalcoholic Fatty Liver Disease: A Narrative Review

**DOI:** 10.3390/nu14214655

**Published:** 2022-11-03

**Authors:** Celeste M. Lavallee, Andreina Bruno, Christopher Ma, Maitreyi Raman

**Affiliations:** 1 Department of Medicine, University of Calgary, Calgary, AB T2N 4N1, Canada; 2 Institute of Translational Pharmacology, National Research Council of Italy (CNR), Via Ugo La Malfa, 153, 90146 Palermo, Italy; 3 Department of Community Health Sciences, University of Calgary, Calgary, AB T2N 4N1, Canada; 4 Snyder Institute of Chronic Diseases, University of Calgary, Calgary, AB T2N 4N1, Canada

**Keywords:** intermittent fasting, time-restricted feeding, intermittent energy restriction, alternate-day fasting, nonalcoholic fatty liver disease

## Abstract

Intermittent fasting is a non-pharmacological dietary approach to management of obesity and metabolic syndrome, involving periodic intervals of complete or near-complete abstinence from food and energy-containing fluids. This dietary strategy has recently gained significant popularity in mainstream culture and has been shown to induce weight loss in humans, reduce gut and systemic inflammation, and improve gut microbial diversity and dysbiosis (largely in animal models). It has been hypothesized that intermittent fasting could be beneficial in the management of nonalcoholic fatty liver disease, given the condition’s association with obesity. This review summarizes protocols, potential mechanisms of action, and evidence for intermittent fasting in nonalcoholic fatty liver disease. It also highlights practical considerations for implementing intermittent fasting in clinical practice. A search of the literature for English-language articles related to intermittent fasting or time-restricted feeding and liver disease was completed in PubMed and Google Scholar. Potential mechanisms of action for effects of intermittent fasting included modulation of circadian rhythm, adipose tissue and adipokines, gut microbiome, and autophagy. Preclinical, epidemiological, and clinical trial data suggested clinical benefits of intermittent fasting on metabolic and inflammatory markers in humans. However, there was a paucity of evidence of its effects in patients with nonalcoholic fatty liver disease. More clinical studies are needed to determine mechanisms of action and to evaluate safety and efficacy of intermittent fasting in this population.

## 1. Introduction

In 2015, the prevalence of overweight and obesity in adults in the United States was 64.2% and 28.3%, respectively [1]. Pathophysiological changes resulting from overweight and obesity lead to metabolic dysfunction, chronic inflammation, and impaired immune system regulation [2]. Obesity and insulin resistance are major risk factors for the development of NAFLD [3]. With a global prevalence rate of close to 30%, NAFLD is the most common cause of chronic liver disease worldwide [4,5], and the number of NAFLD cases globally is projected to increase by up to 56% between 2019 and 2030 [6]. In 2020, an international consensus panel proposed redefining the term “NAFLD” as “metabolic dysfunction-associated fatty liver disease” (MAFLD) to align the disease name closer to its pathophysiology [7]. However, because NAFLD and MAFLD each represent a different patient phenotype, those terms are not interchangeable. As MAFLD has only recently been defined, this review discusses evidence from studies in patients with NAFLD. Lifestyle management, including weight reduction and physical activity, continue to be primary treatment modalities for NAFLD [8]. Weight-loss reduction of ≥10% can decrease hepatic steatosis and result in fibrosis regression [8]. Despite studies demonstrating transformative effects of weight loss on NAFLD and metabolic outcomes, durable weight loss remains elusive for many. In a meta-analysis of 29 long-term weight-loss studies, more than half of lost weight was regained within 2 years, and nearly 80% of lost weight was regained within 5 years [9].

Intermittent fasting (IF) is a dietary intervention that involves periodic intervals of complete or almost complete abstinence from food and energy-containing fluids. The practice of IF has been performed since the time of the earliest civilizations, mainly for religious or cultural reasons [10], and may or may not include energy restriction. Moreover, IF has demonstrated health benefits without weight loss in cancer [11,12], cardiovascular disease [12], and cognitive and brain function [13]. IF has gained popularity with the public [14], with high-profile individuals endorsing its effectiveness, yet guidance for its use and/or implementation is not currently available for patients with NAFLD, highlighting the timely need for evidence-based recommendations for IF in those patients.

Observational studies have reported metabolic benefits of IF, including weight loss [15], improved glycosylated hemoglobin [15], decreased atherogenic risk [16,17], improved circulating cytokines [16,17], and immune cell proliferation [18]. Further, in a systematic review and meta-analysis of five randomized controlled trials (RCTs) comparing IF to energy restriction in patients with type 2 diabetes mellitus and metabolic syndrome, changes observed in glycosylated hemoglobin and fasting plasma glucose were similar between groups; however, IF appeared to have a greater weight-loss benefit (−1.70 kg, 95% confidence interval [CI]: −3.28 to −0.11 kg) [19].

Intervention studies have also reported weight loss [20], improvement in insulin resistance [20], reduced oxidative stress [20,21], improvements in serum cholesterol and triglycerides [21], and decreased levels of systemic inflammatory markers, including tumor necrosis factors (TNF)-α and brain-derived neurotrophic factors [21]. An umbrella review of 11 meta-analyses, including a total of 130 RCTs with comparator groups assigned either continuous energy restriction or regular diet, reported that IF was associated with weight loss; reduced fat mass; and improvements in blood lipids, fasting plasma glucose, fasting insulin, blood pressure, and C-reactive protein [22].

Based on results of observational and clinical studies in other disease states [23,24,25,26,27], it can be postulated that IF would confer metabolic and likely clinical benefits to patients with NAFLD, and clinical evidence is beginning to emerge for use of IF in management of the disease. In this narrative review, we describe current evidence for use of IF in management of NAFLD, as well as potential mechanisms of action, including those related to circadian rhythm, white adipose tissue (WAT), adipokines, the gut microbiome, and autophagy.

## 2. Search Methods

CML and AB conducted a search of the literature for English-language manuscripts in PubMed and Google Scholar. Search terms included “intermittent fasting”, “intermittent AND fasting”, “time-restricted feeding” AND “liver disease”, “nonalcoholic fatty liver disease”, and “NAFLD”. Articles were assessed if they included results of preclinical studies in animal models or of epidemiological or clinical trial studies in humans with NAFLD. Reference lists of identified articles were also assessed to identify additional manuscripts.

## 3. Intermittent Fasting Protocols

Fasting and feeding intervals vary in practice of IF. Various IF protocols are described in Table 1, including time-restricted feeding (TRF), intermittent energy restriction (IER), alternate-day fasting (ADF), 5:2 fasting, and fasting-mimicking diet (FMD).

Specific recommendations regarding diet composition and macronutrient breakdown for patients with NAFLD could not be inferred from existing IF studies, given that IF protocols typically included ad libitum intake during the feeding period, and diet composition and nutrient intake during this feeding period were often not assessed. However, practical, general recommendations for IF implementation for both clinicians and patients are included in Table 2 [28,29].

## 4. Potential Mechanisms of Action for Intermittent Fasting Effects

**Figure 1:**
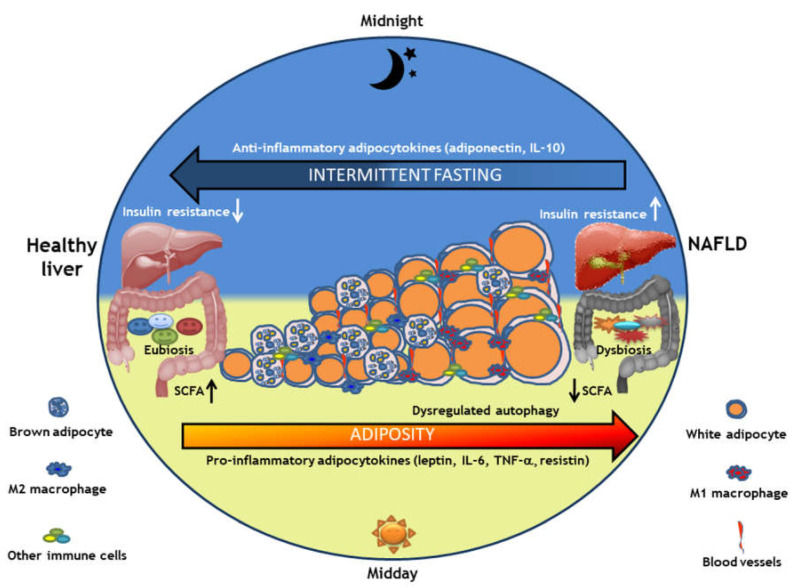
Potential mechanisms of action for effects of intermittent fasting in NAFLD. Obesity is a risk factor for NAFLD [3] and is associated with low-grade inflammation [2,32] marked by presence of increased white adipose tissue, increased pro-inflammatory M1 macrophages, decreased anti-inflammatory M2 macrophages [2], formation of new blood vessels (angiogenesis), increased gut microbial dysbiosis [40], and changes in autophagy-related physiological processes [41]. Intermittent fasting may play a role in circadian rhythm modulation [34,37], adipose tissue plasticity [35,36], adipokine production [23], and gut microbiome [23,33] through increased SCFA production [38,39] and autophagy [31], with potential to reverse inflammation, metabolic dysfunction, and impaired immune system regulation associated with NAFLD. Abbreviations: NAFLD, nonalcoholic fatty liver disease; SCFA, short-chain fatty acids.

IF interventions appeared to confer health benefits independent of energy intake (Figure 1) [2,3,23,30,31,32,33,34,35,36,37,38,39,40,41]. For example, in two separate trials of overweight women, participants lost the same amount of weight during a 6-month period whether they followed an IF intervention or a 25% energy-restricted diet. However, those assigned to the IF group had greater improvement in insulin sensitivity and greater reduction in waist circumference [26,27]. Here, we review physiologic changes induced by IF and their resultant impacts on metabolic health.

### 4.1. Circadian Rhythm

Energy intake plays a fundamental role in controlling eating behavior due to the interaction between the central homeostatic and non-homeostatic (hedonic) systems [42]. Balance between homeostatic and hedonic eating behaviors is not only influenced by volume and composition of the diet, but also by timing and rhythmicity of food ingestion. Furthermore, circadian rhythm affects gut function as well as composition and interactions of the microbiome with the gut [33]. Circadian rhythm represents all physiological processes involved in a period of 1 day, such as sleep/wake cycle, vital signs, hormone secretion, cognitive performance, and mood regulation. Of interest: limiting time of food consumption appeared to readjust the circadian clock, implying that meal timing affects metabolism [43]. While the circadian system modulates both insulin and glucagon by controlling production and secretion, the system itself is regulated by numerous factors, including food intake. Although insulin production and secretion are maximal in the evening, around 5 p.m., and lowest in the early morning, at 4 a.m. [37], eating patterns alter blood nutrient levels and may override circadian patterns of hormonal release, which may have implications on various physiological processes.

For example, in a small study of 23 adults who fasted during the daytime for the month of Ramadan, evening cortisol levels were higher compared to those observed during a non-fasting month [44]. An increase in evening cortisol coincided with increased insulin levels in the evening and increased blood glucose levels in the morning. Another small study of 11 overweight adults participating in a 4-day randomized crossover study of TRF in which food was consumed either between 8 a.m. and 2 p.m. (early fasting) or 8 a.m. and 8 p.m. (control) demonstrated improved mean 24-h glucose levels and induced broad changes in circadian clock gene expression, as well as expression of hormones and genes related to longevity and autophagy in the early fasting group as compared to the control, implying anti-aging effects [34]. In a further delve into molecular mechanisms of IF, the hepatic macrophage glucocorticoid receptor was recently identified as an important component of fasting-induced macrophage secretion of inflammatory cytokines, and its relationship to influence of ketogenesis in hepatocytes was demonstrated [45].

In relation to NAFLD, Kettner et al. demonstrated that disruption of the circadian rhythm in jet-lag-induced mice fed ad libitum resulted in metabolic syndrome and liver injury, progressing from NAFLD to nonalcoholic steatohepatitis (NASH) and fibrosis, similar to those seen in mice lacking the circadian genes Bmal1, Per1; Per2, or Cry1; Cry2 and therefore lacking a circadian clock [46]. In a separate study, Chaix et al. demonstrated that weight gain and hepatic steatosis were prevented by TRF within a 10 h period in mice that were jet-lagged, similar to in mice without a circadian clock, also suggesting that eating patterns might override circadian rhythm [47].

### 4.2. White Adipose Tissue Browning

Historically, adipose tissue has been viewed as an inert, passive pool for energy storage, and while that is accurate, its endocrine functions have also been widely established [48]. Adipose tissue has been demonstrated to express and secrete a wide variety of bioactive peptides, collectively referred to as adipokines. Examples of adipokines include leptin, adiponectin, TNF-α, interleukin (IL)-6, and resistin, among others. Adipokines may act either locally, using paracrine or autocrine signaling, or systemically, through neurocrine and endocrine pathways. Adipose tissue may be subdivided into WAT and brown adipose tissue (BAT), and further into visceral and subcutaneous depots of fat. Adipose tissue sub-types and compartments have varying impacts upon health and disease.

The main role of WAT is storage of energy as triglycerides [49], whereas BAT is involved in thermogenesis [49,50], which may counteract the effects of increased energy intake and promote weight loss. Consequently, browning of WAT may represent a promising therapeutic target for obesity and metabolic diseases. A preclinical study performed in mice reported that IF promoted WAT browning and ameliorated obesity, insulin resistance, and hepatic steatosis by altering the gut microbiome [36]. In another pre-clinical study, isocaloric IF improved metabolic homeostasis through adipose thermogenesis by WAT browning due to M2 anti-inflammatory macrophage activation and increased vascular endothelial growth factor expression [35].

### 4.3. Adipokines

Adipose tissue is comprised of several different types of cells, including fibroblasts, endothelial cells, and different types of immune cells. In the context of obesity, immune-cell composition of adipose tissue undergoes major changes, with abnormal adipokine and chemokine production as well as activation of inflammatory signaling pathways [51]. Adipokines exert anti-inflammatory or pro-inflammatory effects in different organs. Generally, pro-inflammatory adipokines include leptin, visfatin, IL-6, TNF-α, resistin, and fatty acid binding protein 4, whereas examples of anti-inflammatory cytokines include adiponectin, IL-10, omentin-1, vaspin and apelin [52].

With obesity, greater secretion of pro-inflammatory adipokines (including leptin, IL-6, TNF-α, and resistin) was observed, affecting satiety and lipid metabolism, along with a decrease in anti-inflammatory and insulin-sensitizing cytokines such as adiponectin and IL-10 [51]. Of fat depots, visceral adipose tissue (VAT) was the major determinant of inflammatory cytokine production, and decreases in VAT were associated with improved inflammatory outcomes [53]. Even a decrease in weight as low as 2.5% reduced fat mass after 3 weeks of IF in subjects without obesity [54] and reduced VAT area after 8 weeks of IF in subjects with obesity [55]. This is of particular relevance in NAFLD, as VAT area is associated with greater risk of developing NAFLD [56], with fibrosis [57,58], and with lower likelihood of disease improvement [56]. A recent narrative review described the link between adipokines and NAFLD and progression to NASH and cirrhosis. Low levels of leptin with high levels of adiponectin were protective against hepatic steatosis, whereas high levels of leptin and resistin acted to increase hepatic fibrosis [59]. Those effects highlighted a plausible role for alteration of adipokine secretion, through interventions such as IF, to limit the progression of liver disease.

In the context of intestinal inflammation, it has been proposed that adipokines arising from VAT, such as leptin, may increase permeability of mucosa, thereby facilitating bacterial translocation and the inflammatory process [60,61]. Decreased leptin with increased adiponectin levels has been observed in humans who fasted on alternate days for 12 weeks [24]. However, in a different study, leptin levels decreased in subjects with overweight or obesity who followed an ADF protocol, but adiponectin levels did not change, nor did other biomarkers of inflammation, including resistin, IL-6, or TNF-α [62].

Similarly, in a pilot RCT in patients with multiple-sclerosis that compared ADF with energy intake restricted to 500 kcal/day on fasting days to ad libitum diet for 15 days, reduced leptin levels were found after ADF, but no differences were noted in adiponectin levels between IF and ad libitum diet [23]. Differences in sample size, adherence, and drop-out may have accounted for these divergent results. Further studies are warranted to better delineate the specific mechanistic effects of fasting on adipokines, which could potentially be useful for biomarkers of fasting and inflammation in clinical practice.

### 4.4. Adipose Tissue–Gut Microbiome Axis

The gut microbiome is increasingly recognized for its role in systemic metabolism [63,64]. Gut microbiota mediate hepatic production of triglycerides and promote storage of triglycerides in adipocytes by suppressing expression of lipoprotein lipase inhibitors [64]. Gut microbial dysbiosis, characterized by decreased microbial gene richness and altered abundance of microbial groups when compared to those of people of normal weight, was noted in individuals with obesity [65]. Specifically, an increased Firmicutes to Bacteroidetes ratio, increased abundance of *Lactobacillus* and *Bifidobacterium,* and decreased abundance of *Akkermansia muciniphila* were observed in individuals with obesity [66,67,68].

Notably, gut microbial dysbiosis seemed to contribute to the conversion of choline into methylamines and to alter bile acid metabolism, both of which can induce hepatic inflammation, leading to NAFLD [69]. Therefore, improving microbial dysbiosis may be an important mechanism in reducing hepatic inflammation and limiting the progression of NAFLD. Interestingly, studies in humans have also noted changes in the microbiome during IF. Results from a pilot observational study of Ramadan fasting demonstrated an increase in *A. muciniphila* and *Bacteroides fragilis*, with an amelioration of lipid and glucose profiles [70]. Furthermore, a recent clinical review described the positive association of *A. muciniphila* with IF-mediated improvements in host energy metabolism and circadian rhythm [71].

Gut microbiota may contribute to obesity pathogenesis through modulation of energy metabolism via enabling of energy extraction from otherwise indigestible foods, which may provide as much as 10% of the daily energy requirement [72]. Further, lipopolysaccharide from the outer membrane of Gram-negative bacteria may translocate through the intestinal barrier and contribute to systemic inflammation [73]. Metabolic endotoxemia has been shown to influence onset and progression of insulin resistance and metabolic disease, which is of relevance to NAFLD pathogenesis [74]. Finally, the gut microbiome contributes to appetite by modifying gut hormones released by the enteroendocrine cells, such as ghrelin, peptide YY, leptin, and glucagon-like peptide 1.

Preclinical studies have demonstrated the ability of IF to beneficially modulate the gut microbiome. IF for 28 days in mouse models of type 2 diabetes mellitus improved intestinal permeability, decreased plasma lipopolysaccharide, improved gut microbial diversity, and increased abundance of several bacteria at the genus level, including butyrate producer Odoribacter [75]. Moreover, ADF compared to isocaloric intake in mice induced WAT browning, weight loss, and changes in gut microbiota, including an increase in Firmicutes to Bacteroides ratio [36]. Of note is that microbiota-depleted mice were resistant to IF-induced WAT browning, while fecal microbiota transplantation from IF-treated mice to microbiome-depleted mice activated WAT browning, providing a potential gut-microbiota-driven mechanism to explain WAT browning and a rationale for treating metabolic diseases.

### 4.5. Autophagy

Autophagy is a vital catabolic process by which cells degrade and recycle endogenous and exogenous components to maintain cellular homeostasis [76]. It facilitates eradication of damaged cell organelles or unused proteins, and is stimulated by various conditions of stress, including starvation. Autophagy is necessary when a cell is deprived of compounds needed for survival. Therefore, calorie restriction is the most robust modifiable inducer of autophagy, and, interestingly, nutrient depletion or limitation is associated with longevity [77]. Alternatively, dysregulated autophagy is associated with several chronic disorders, including metabolic diseases [78].

Given the potency of calorie restriction to induce autophagy, it can be posited that IF could attenuate this dysregulation. Indeed, mice that had fasted in an Alzheimer’s disease model showed an increase in number and size of autophagosomes in neurons [31]. Another preclinical study in a mouse model of Charcot–Marie–Tooth syndrome demonstrated five months of IF increased expression of autophagy-associated proteins, ATG7, and microtubule-associated protein 1 light chain 3, as well as decreased levels of p62 protein, collectively suggesting increased autophagy [79].

## 5. Studies of Intermittent Fasting in Nonalcoholic Fatty Liver Disease

Lifestyle modification consisting of dietary intervention and physical activity remains the only therapeutic option for most patients with NAFLD [3,80,81]. However, there was limited evidence as to which dietary interventions are most effective at improving or resolving NAFLD [3,8,80,81]. There were few head-to-head RCTs that evaluated the efficacy of specific dietary interventions on hepatic, metabolic, and weight-loss outcomes in patients with NAFLD, and it is widely accepted that more high-quality dietary studies are needed to confirm a successful approach [82].

Preclinical evidence in NAFLD animal models supported further clinical inquiry into IF as a potential therapy [83,84], and observational [85] and retrospective [86] studies on the effects of fasting during Ramadan supported use of IF for management of NAFLD. To date, three RCTs have evaluated the use of IF in patients with NAFLD [87,88,89].

In a 12-week RCT, 271 adult patients with NAFLD were randomly assigned to an ADF, a TRF, or a control group [87]. Patients in the ADF group consumed 25% of their energy needs on fasting days and ate ad libitum on non-fasting days. Patients in the TRF group ate ad libitum during any consecutive 8 h window of their choice each day. Patients in the control group consumed 80% of their energy needs despite not being given guidance or restrictions regarding their usual intake. Energy intake on feeding days was not different between the groups. Composition of macronutrient intake was not reported. At 12 weeks, patient-reported dietary adherence was 97.5%, with no significant differences between the three groups. Body weight decreased significantly from baseline in both the ADF (−5.4 ± 0.7%) and TRF (−4.3 ± 0.9%) groups as compared to the control (−2.54 ± 0.9%) after 12 weeks, which was attributed largely to loss of fat mass. Compared to controls, total cholesterol decreased with ADF but not TRF, while triglycerides decreased with both IF protocols. No differences were found in fasting insulin, glucose, or blood pressure, although study duration was quite short. Liver stiffness, assessed by FibroScan^®^, was not different within or between groups after 12 weeks, and no other liver-related biomarkers, including liver enzymes or sonographic features of steatosis, were assessed in the study.

However, in another 8-week RCT, liver steatosis and fibrosis, assessed using ultrasound and two-dimensional shear wave elastography, respectively, decreased significantly with ADF as compared to the control [88]. In this study, patients with NAFLD were randomly assigned 3:1 either to follow an ADF (*n* = 33) that consisted of 30% of energy needs on fasting days and ad libitum consumption on non-fasting days or to maintain habitual intake (control; *n* = 10). Overall energy and composition of macronutrient intake were not assessed on feeding days. ADF also reduced body weight, body mass index (BMI), alanine transferase, and aspartate transferase as compared to controls.

Improvements were also seen with IF in another 12-week RCT of adults with NAFLD [89]. Patients were randomly assigned to receive either 5:2 IER (*n* = 25); a low-carbohydrate, high-fat (LCHF) diet (*n* = 25); or standard-of-care (SoC) as a control (*n* = 24). The 5:2 IER protocol consisted of fasting on two non-consecutive days per week, wherein patients restricted energy intake on fasting days to 500 and 600 kcal/day for women and men, respectively. In both the 5:2 IER and LCHF groups, women and men consumed an average daily caloric intake of 1600 and 2000 kcal/day, respectively, over 7 days. The 5:2 diet was based on Nordic Nutrition Recommendations [90] and consisted of 45–60%, 25%, and 10–20% energy from carbohydrates, fat, and protein [89]. The LCHF diet consisted of 5–10%, 50–80%, and 15–40% of energy from carbohydrates, fat, and protein, respectively. Patients in the SoC arm received advice to follow a healthy diet, increase intake of unsaturated fat while reducing intake of saturated fat, reduce sweets, limit portion sizes, and eat three meals per day, although adherence to these recommendations was not assessed. Change in liver steatosis was measured by magnetic resonance spectroscopy and liver stiffness was assessed by radiology or FibroScan^®^. Patients following either the 5:2 IER or LCHF diet achieved reductions in steatosis and body weight (−7.4%, 95% CI: −8.7 to −6.2% for IER and −7.7%, 95% CI: −10.0 to −5.4% for LCHF) as compared to SoC (−2.6%, 95% CI: −3.7 to −1.5%), but only patients in the 5:2 IER and SoC groups attained reduced liver stiffness.

Collectively, these results suggest that IF may be a promising therapy for reducing body weight, liver steatosis, and liver stiffness in patients with NAFLD. Given differences in methodologies, whereby not all studies assessed or reported differences in total energy or macronutrient intake, it is unknown whether differences in outcomes between the groups were related to timing of IF protocol, related energy restriction, or related changes in diet composition. More and longer-term studies are needed to assess comparative efficacy of various IF protocols versus emerging pharmacotherapies, adherence to IF, and long-term responses.

## 6. Potential Risks of Intermittent Fasting

Despite potential benefits of IF, the practice may not be appropriate for all patients or populations. First, while evidence on the effects of fasting on mothers and offspring during pregnancy is emerging, it is conflicting, and long-term effects are not known. Therefore, IF during gestation should be advised against until more substantial evidence is available. Second, although IF appears safe for people with type 2 diabetes [19,91], caution is warranted for anyone taking glucose-lowering medication. These patients should be followed closely by their healthcare providers to ensure proper monitoring and adjustments to their glucose-lowering medications, as needed. Finally, all patients should be made aware of potential physical or psychosocial effects of IF. Side effects that have been reported in studies of IF include reduced energy levels, headache, presyncope, decreased concentration, mood swings or bad temper, feeling cold, constipation, bad breath, and preoccupation with food [26,27].

## 7. Conclusions

Obesity has an integral role in development of NAFLD. Sustainable weight loss remains challenging for most patients, and there is rationale from preclinical studies as to potential benefits and mechanisms of IF to ameliorate metabolic disturbances, influence the gut microbiome and bacterial translocation, and contribute to modest weight loss. While epidemiological and clinical trial data were encouraging for clinical benefits of IF on various metabolic and inflammatory markers in humans, there were limited data for effects in human subjects with NAFLD. High-quality clinical studies of patients with NAFLD are needed to identify mechanisms of action as well as effectiveness, safety, and efficacy of IF in this population. Priorities for future research include the impact of IF on long-term natural history, fibrosis regression, potential effect modification between diet and pharmacologic therapy, and comparative efficacy of different IF protocols. Furthermore, those studies should also evaluate dietary intake during feeding periods, measured through validated food frequency questionnaires and/or 24 h recalls, to inform an optimal dietary pattern in the setting of IF interventions.

## Figures and Tables

**Table 1:** Intermittent fasting protocols.

Type of Intermittent Fasting Protocol	Description of Intermittent Fasting Protocol
Time-restricted feeding	Commonly entails a daily fast for 12 to 20 h
Intermittent energy restriction	Involves an energy-restricted fast during intermittent periods, during which some energy-laden foods or liquids are consumed
Alternate-day fasting	Involves fasting for 24 h, followed by a regular eating pattern for the following 24 h
5:2 fasting	Consists of fasting on two non-consecutive days for every five days of regular intake
Fasting-mimicking diet	Includes some energy-laden liquids

**Table 2:** Practical recommendations to implement intermittent fasting in patients with NAFLD.

Steps	Recommendations for Each Step
Step 1: Determine if patient is overweight or obese	• Assess weight, anthropometrics, BMI, waist circumference, and metabolic comorbidities
	• Rule out edema or ascites
Step 2: Assess the patient’s history with weight-management strategies	• If patient is naïve to weight management, propose all dietary options, including IF
	• If patient has experience with dietary weight-loss interventions that were ineffective, propose IF as an option
Step 3: Screen for risk of malnutrition	• Screen for malnutrition risk using the abridged PG-SGA ^a^
	• Consider using the NIAS ^b^ to rule out avoidant and restrictive food behaviors that may increase the risk of malnutrition risk from an IF intervention
Step 4: Support the patient as a partner in their journey	• Empower the patient with options for weight management, including but not limited to intermittent fasting. Other options include calorie-restriction, physical activity, and, when appropriate, anti-obesity medications and bariatric surgery
Step 5: Determine the IF protocol that appeals to the patient, matches their preferences/lifestyle, and is most likely to result in long-term adherence	• If the patient chooses IF, review different protocols and encourage the patient to consider factors such as sleep/wake cycles/times, shift work, current patterns of eating, diabetes, other comorbidities, etc.
	• There was insufficient evidence to recommend one IF pattern over another; the choice should be determined based on lifestyle and other comorbidities (e.g., ADF may not be suitable for a patient treated with glucose-lowering medications)
Step 6: Facilitate IF adherence and nutritional adequacy	Provide recommendations to:
	• Optimize hydration with water
	• Limit sugar and artificially sweetened beverages
	• Choose high-fiber foods, such as fruits, vegetables, beans, and lentils, as tolerated
	• Include protein sources such as fish, poultry, eggs, beans, lentils, and cheese
	• Include 2–3 Tbsp of healthy fats each day, such as nuts, seeds, and olive oil
**Step 7**: Monitor for side effects and malnutrition While malnutrition and other adverse events are not expected, patient and clinician should both monitor for adverse outcomes	Monitor for:
	• Malnutrition risk (using the abridged PG-SGA^a^)
	• Fatigue, headache, and muscle cramps (ensure adequate intake of electrolytes, especially during the fasting period)
	• Excessive weight loss (greater than 1 kg per week)
	• Micronutrient deficiencies (iron, folate, vitamin B12)

^a^ Abridged patient-generated subjective global assessment [28]. ^b^ Nine item avoidant/restrictive food intake disorder screen [29]. Abbreviations: ADF, alternate-day fasting; BMI, body mass index; IF, intermittent fasting; NAFLD, nonalcoholic fatty liver disease; NIAS, nine item avoidant/restrictive food intake disorder screen; PG-SGA, patient-generated subjective global assessment; TRF, time-restricted feeding.

## Data Availability

No new data were created or analyzed in this study. Data sharing is not applicable to this article.
